# PROTOCOL: Understanding Intergenerational Programmes to Improve the Psychosocial Health and Well‐Being of Older Adults in Residential Aged Care: A Rapid Realist Review Protocol

**DOI:** 10.1002/cl2.70023

**Published:** 2025-04-08

**Authors:** Lysha Z. Y. Lee, Patricia Nicholson, Katrin Gerber, Ramona Naicker, Alison M. Hutchinson

**Affiliations:** ^1^ School of Nursing & Midwifery, Centre for Quality and Patient Safety, Institute for Health Transformation Deakin University Melbourne Victoria Australia; ^2^ National Ageing Research Institute Melbourne Victoria Australia; ^3^ Faculty of Medicine, Dentistry and Health Sciences The University of Melbourne Melbourne Victoria Australia; ^4^ Library, Outreach and Scholarly Services Deakin University Melbourne Victoria Australia; ^5^ Barwon Health Geelong Victoria Australia

## Abstract

This is the protocol for a Campbell systematic review. The main objective of this realist review is to gather, critically appraise, and synthesize evidence to identify what works, for whom, in what circumstances, and how non‐familial intergenerational programs involving pre‐school children work to improve the psychosocial well‐being of older adults living in residential aged care settings.

## 1 Background

Demographic ageing poses a global challenge for the aged care sector, with significant implications for health and wellbeing, social services and the economy. Developed nations are restructuring their social systems to promote ageing at home; however, a significant number of older adults still require specialised care in residential aged care (RAC) homes and will continue to have such needs into the foreseeable future (National Institute on Aging 2017; Parker 2022).

Yet, the prevalence of poor mental health and reduced psychological well‐being among older adults in RAC settings is significant, particularly when compared to their peers living in the community. Authors of a systematic review identified that between 3.2 to 20% of 5927 residents had a diagnosed anxiety disorder, compared to estimates of 1.4 to 17% in the community (Creighton et al. 2016). In a retrospective cross‐sectional study involving 430,000 older Australians living in permanent residential aged care, 57.8% had at least one mental health disorder, with depression (46.2%) being the most frequently reported (Amare et al. 2020). In the United Kingdom, people living in RAC facilities exhibited depressive symptoms at double the rate of their community‐dwelling counterparts (Matthews et al. 2016). Mezuk and colleagues (2014) reported that suicidal ideation was prevalent among up to one‐third of aged care residents, a figure four times higher than that observed in older adults living independently in the community.

An individual's transition to a RAC home is commonly associated with various losses, such as independence and autonomy. Additionally, the older person often contends with social isolation from their community and, in certain instances, family (Kelly et al. 2021; Riedl et al. 2013; Zamanzadeh et al. 2017). Despite living in a communal environment, many individuals in these facilities are socially isolated (Nikmat et al. 2015). The authors of a meta‐analysis estimated the prevalence of moderate loneliness in RAC ranged from 31% to 100%, while severe loneliness ranged from 9% to 81% (Gardiner et al. 2020).

An increasing body of empirical evidence demonstrates that social isolation and loneliness are risk factors for public health issues, including an increased risk of poor physical and mental well‐being, cognitive decline, dementia (Alun and Murphy 2019; Lara et al. 2019; Penninkilampi et al. 2018; Siette et al. 2020), and premature mortality (Ong et al. 2016; WHO 2021). Conversely, having extensive social networks is associated with cognitive health and subjective well‐being (Siette et al. 2020). Authors of a meta‐analysis highlighted that across 5115 older adults living in RAC facilities, the prevalence of moderate and severe loneliness ranged from 31 to 100%, and 9 to 81%, respectively (Gardiner et al. 2020). Residents used descriptors including “trapped”, “stuck”, “confined”, “isolated”, and “discouraged” to articulate their sentiments about institutional life (Choi et al. 2009). While most service providers recognise that aged care providers should focus on wellness, rehabilitation and reablement (Lewin et al. 2013), the psychosocial needs of older persons in RAC are often neglected during care provision due to scarcity of resources and competing medical needs (Ibrahim et al. 2020). Thus, offering innovative solutions to enable community engagement, reduce social isolation, and promote quality of life and well‐being for older people living in RAC is imperative and needs to be prioritised.

## 2 Intergenerational Practice

Intergenerational practice is a human service strategy that brings two or more generations together to engage in purposeful, mutually beneficial activities. The broad aim of these programs is to foster more positive intergenerational attitudes and build more cohesive communities (Beth Johnson Foundation 2009). Over recent decades, intergenerational programs (IGPs) have been developed to address various issues and improve social, emotional, and behavioural outcomes (Laging et al. 2022). More recently, there has been a shift towards addressing ageism, social isolation, loneliness, and generational segregation. Whilst familial IGPs focus on relationships within families (e.g., grandparent‐grandchild dyads), most of the recent literature explores non‐familial IGPs, giving individuals who are not biologically related a platform to connect. Factors such as changing family structures, migration, and the increased use of child and aged care are making geographically distant grandparents increasingly common (Warren et al. 2020). Non‐familial intergenerational practice, therefore, offers a fitting solution to bridge the intergenerational gaps that exist today.

A considerable number of primary and secondary studies showed the effectiveness of IGPs in various settings (Campbell et al. 2023; Coon et al. 2022; Gualano et al. 2018; Krzeczkowska et al. 2021; Peters et al. 2021; Steward et al. 2021; Tsiloni et al. 2023). The most investigated impacts on older adults include psychological and social health and well‐being outcomes. Zhong and colleagues (2020) found that social interactions with young children (kindergarten to third graders) had a significant and broad range of health benefits for older adults. This was corroborated by the findings of Krzeczkowska and colleagues (2021) in relation to psychosocial health, social relationships and activity, and cognitive function. Program‐based intergenerational interactions were reported to have significant health‐related benefits for older adults (Gualano et al. 2018; Teater 2016; Zhong et al. 2020).

While much scarcer than reported benefits, some negative effects of intergenerational interventions have also been reported. Authors of a mapping review and evidence gap map (Campbell et al. 2023) reported potential downfalls, including: the burden of program coordination; mismatches in participant pairing; feelings of exclusion; and the impact that loss and death might have on participants. Additional challenges included participants’ disliking activities in the program and the risk of reinforcing negative age stereotypes (Peters et al. 2021).

## 3 Rationale for a Realist Review

Despite the number of articles published in this field, all reviews highlight two main concerns regarding the state of the literature: small sample sizes and heterogeneity between studies. A large proportion of IGP studies were conducted with small samples due to the nature of the intervention (De Bellis et al. 2022; Jarrott 2011; Laging et al. 2022; Lee et al. 2020; Lu et al. 2022; Wendland & Parizet 2022). As a complex intervention with diversity across settings, program designs, sample sizes, evaluation methodology and outcome measures, it is challenging for researchers to synthesise, generalise and interpret findings using traditional methods.

Another knowledge gap researchers have identified is a lack of transparency regarding the mechanisms underlying programs linked to change. Consequently, a limited understanding of the components that constitute meaningful intergenerational engagement remains (Laging et al. 2022). The common sentiment amongst researchers appears to be that “intergenerational activities can enrich the lives of participants, but the mechanisms are poorly understood” (De Bellis et al. 2022, p. 380). Studies to date have primarily focused on the outcomes of IGPs, providing limited understanding of how and why they result in different outcomes for different individuals, in different settings, or for which populations they are most suited (Fakoya et al. 2021). Without uncovering and identifying the mechanisms responsible for intended and unintended program outcomes, practitioners face challenges in determining the root cause when desired impacts are not realised. This ambiguity makes it challenging to ascertain whether the issue lies in implementation or theoretical failure – i.e., employing inappropriate strategies in an intergenerational context ‐ or that intergenerational strategies were not a suitable approach to address the identified needs (Jarrott et al. 2021; Stame 2010). While earlier studies are insightful and necessary, the evidence base does not adequately provide intergenerational practitioners with the information needed to successfully implement IGPs in their own contexts.

Lastly, whilst there are several theories that intergenerational practitioners and academics refer to, a theoretical framework that can underpin IGP development and implementation has not been developed. This issue has been raised by scholars for over two decades:

“We continue to mount new intergenerational programs, but without an adequate conceptual framework to guide the design and implementation of these efforts.”

– (VanderVen 2004) (p. 76)

“While theories of the life course and of human development in various phases of the life course abound, the intergenerational field still needs its own conceptual framework to communicate its own identity and serve as a means for interacting with these and other related fields.”

– (Vanderven 2011) (p. 22)

“It is also important to note that a number of other researchers and commentators over the past decade have continued to alert us to the fact that, without theory, phenomena such as intergenerational practice or relationships cannot be fully understood (Bernard, 2006; Granville, 2002; Jarrott 2011; Kuehne, 2003a; VanderVen 2004, 2011). Likewise, it is important to root practice in theory (Lawrence‐Jacobson, 2006) as it can help us in determining the key aims of intergenerational projects and programs.”

– (Kuehne & Melville 2014) (p. 318)

Jarrott's (2011) content analysis of publications in the field highlighted that out of 127 studies, only 35% referred explicitly to one or more theories, whilst 39% made no reference to a theory. At the same time, it has been found that theory‐informed IGPs are more effective in achieving intended outcomes (Jarrott & McCann 2013; Jarrott & Smith 2011), demonstrating the potential impacts of interventions underpinned by appropriate theory.

Social interventions like non‐familial IGPs can be complex, multifaceted and dynamic. Given the heterogeneity across existing studies, traditional systematic review methodologies are not likely to yield the most valuable information in terms of transferability. On the other hand, the realist review methodology is particularly suitable for evaluating complex service interventions (Pawson et al. 2005). The theory‐driven approach also addresses the lack of program theory development in the field. The reviewers determined the suitability of this approach by comparing the nature of complex service interventions with those of IGPs (Table 1).

**Table 1:** List of criteria to determine if non‐familial intergenerational programmes are complex service interventions (CSIs) and appropriate for realist synthesis.

Defining features of CSIs (Pawson et al. 2005)	Non‐familial intergenerational programmes (IGPs)
1. CSIs are **theories**–a hypothesis that postulates: ‘If we deliver a programme in this way/we manage services like so, then this will bring about some improved outcome’.	IGPs can take on hypotheses of, for example, ‘If we include an intergenerational component to programmes, then this will bring about improved psychosocial health and well‐being for the participating residents’.
2. CSIs are **active**–they achieve their effects via the active input of individuals, human volition; thus, at least part of the explanation will be in terms of the reasoning and personal choices of different actors and participants.	IGPs achieve varying effects depending on participants' (young, old) willingness to participate in activities and the degree to and way in which they participate. It is also dependent on the active input of others such as facilitators or staff, resources provided by executives, etc.
3. Intervention theories have **long journeys–**they begin in the heads of programme architects → hands of practitioners/managers → hearts and minds of participants; the success of an intervention depends on the cumulative success of the entire sequence of these mechanisms.	IGPs in residential aged care settings begin as ideas of programme architects (e.g., executive stakeholders), then are shared with those ‘on the ground’ (e.g., facility staff, practitioners), which are then implemented and introduced to participants (e.g., residents, children).
4. Implementation chains of CSIs are **non‐linear** and may **go into reverse.**	The influence of various parties (executives/management, facility staff and practitioners, participants, participants' families) involved in IGPs may affect implementation in different ways.
5. CSIs are **fragile creatures, embedded in multiple social systems**–highlighting the influence of **context** (e.g., organisational culture and leadership, resource allocation, staffing levels and capabilities, interpersonal relationships, competing local priorities & influences).	IGPs are embedded in multiple social systems, including the organisational culture and leadership of both the aged care and education facility, staffing levels and training of staff training for IGPs, interpersonal relationship amongst participants and/or staff, competing local priorities (e.g., clinical care)–which can all influence the effectiveness and implementation of an IGP.
6. CSIs are **leaky** and **prone to be borrowed**–expect the same intervention to be delivered in a mutating fashion shaped by refinement, reinvention and adaptation to local circumstances.	As seen in the literature, IGPs are highly flexible interventions that vary in characteristics such as type of activities, duration, frequency, space, interaction ratios and so on, depending on the local circumstances and context.
7. CSIs are **open systems that feedback on themselves**–as they are implemented, they change the conditions that made them work in the first place; learning occurs that alters subsequent receptivity.	For example, with increased experience, observation and learning, staff involved in IGPs learn the needs of participants and improve their intergenerational facilitation skills and programming, improving the interactions amongst participants and thereby increasing intervention effectiveness.

## 4 Objectives

The review is designed to explore: What works, for whom, in what circumstances, and how do non‐familial intergenerational programs involving pre‐school children work to improve the psychosocial well‐being of older adults living in RAC settings? The aim of the review is to test, validate and refine initial program theories to gain a comprehensive understanding of the mechanisms underlying these programs. Specifically:

- To identify the mechanisms and resources of IGPs that are likely to improve the psychosocial well‐being of older adults in RAC settings.
- To develop a better understanding of the contextual factors that contribute to the success of IGPs in improving psychosocial well‐being of older adults in RAC settings.
- To develop explanatory program theories that can inform the design and implementation of IGPs in RAC settings.

It is anticipated that this evidence synthesis will not only impact the practical decisions of practitioners and providers. Developed theories can be further tested and refined in future research, as well as utilised in developing intergenerational policies nationally and internationally. This review specifically focuses on IGPs that are (a) non‐familial, involving individuals who are not biologically related; and (b) involve older adults with preschool children, thus adjacent (or skipped) generations at minimum.

## 5 Methods

### 5.1 Rapid Realist Review

A rapid realist review, a knowledge synthesis process that prioritises practical and context‐specific explanations for what works within specific parameters, maintaining the fundamental elements of the realist philosophy (Ní Shé et al. 2018; Saul et al. 2013), will be conducted. Rapid realist reviews prove particularly valuable in the initial stages of a multi‐phase project, especially when there is a need for fast adaptation of findings and in circumstances of constrained time and resources (Ní Shé et al. 2018). This is the case for this review, as the results of the review will inform a realist evaluation.

**Figure 1:**
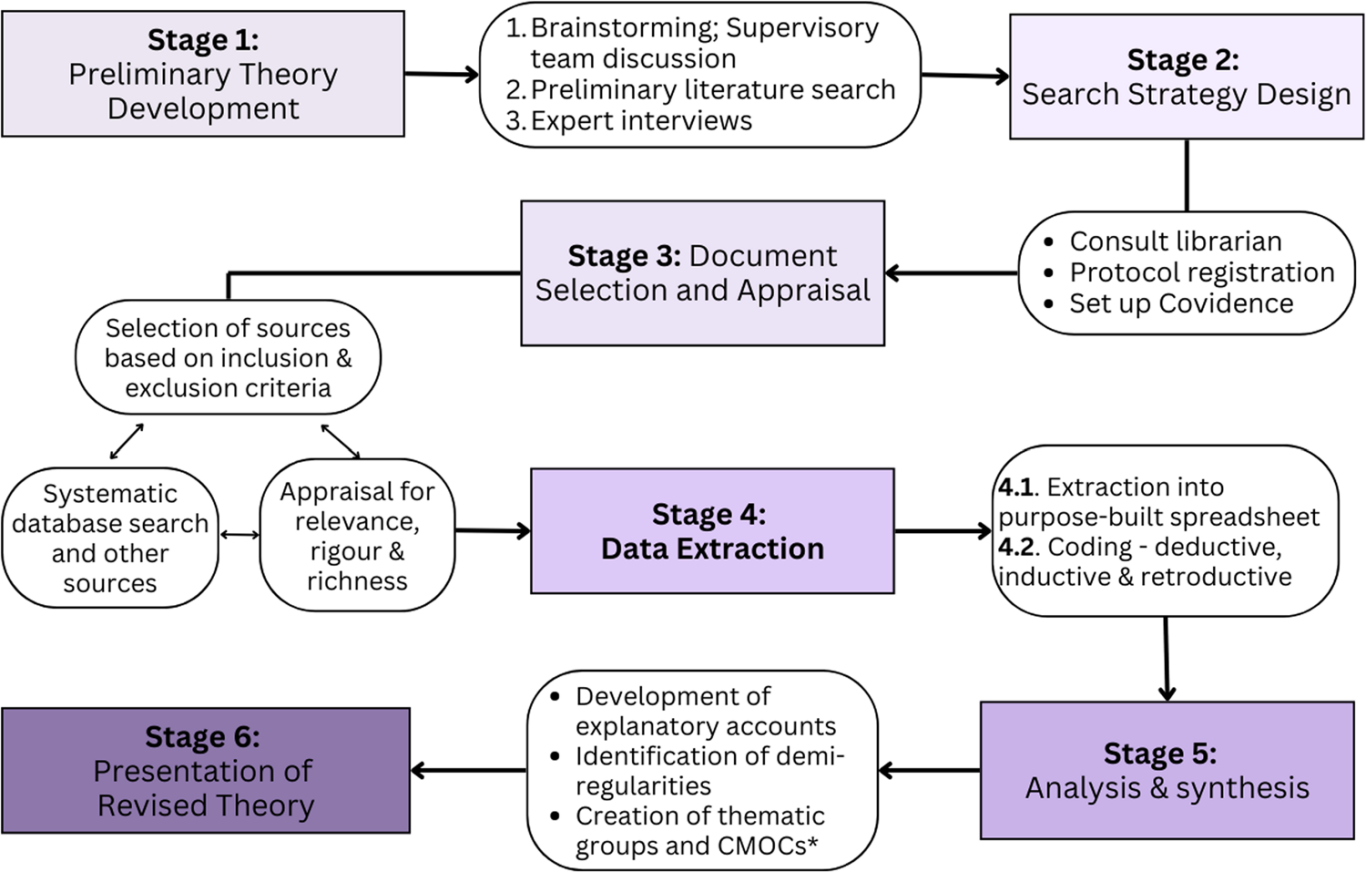
Sequence of stages of the rapid realist review. *CMOCs: Context‐Mechanism‐Outcome Configuration.

This rapid realist review will follow six steps recommended by Pawson et al. (2005) and Wong et al. (2013), which are compatible with those proposed by Saul et al. (2013): (1) preliminary theory development; (2) search strategy design; (3) document selection and appraisal; (4) data extraction; (5) analysis and synthesis; and (6) presentation of revised theory (Figure 1). Conducted over a 9‐month period, this rapid realist review forms a preliminary phase to a realist evaluation that will be undertaken at an intergenerational shared site facility (early childcare learning centre and residential aged care facility) in Australia. The PRISMA‐P checklist (Moher et al. 2015) was used for this protocol (see Supporting Information S1).

#### 5.1.1 Stage 1: Preliminary Theory Development

The first step of a realist review is to make programme theories explicit. This involves clarifying underlying assumptions of how an intervention is meant to work in relation to the expected impacts (Pawson et al. 2005). The development of a priori theories, also known as initial programme theories (IPTs), was an iterative, three‐part process involving (i) brainstorming, retroduction and hunches; (ii) a scoping search of the literature on the topic of enquiry; and (iii) drawing from expert interviews.

The first version of IPTs was developed based on reviewer L.Z.Y.L.'s experiential and professional knowledge as an IGP researcher and facilitator. The following guiding questions were used to brainstorm and think retroductively: What is the logic of IGPs? What is the best explanation as to how IGPs work? What are the underlying assumptions that suggest that IGPs are a good idea?

The second iteration of IPTs was drafted following a preliminary search of the literature from September to October 2023. Databases searched for peer‐reviewed literature included PsycINFO, CINAHL and Google Scholar. Google was searched for grey literature and other relevant documents (e.g., organisational and governmental reports). As this was a broad sweep of the literature, key search concepts were (non‐familial) ‘intergenerational programs’ and ‘older adults’. Snowball searching and reference list tracking were also used. At this point, general abstract and tacit theories non‐specific to IGPs involving preschool children (primarily from systematic reviews) were referenced to develop the IPTs further.

The final iteration of IPTs was developed based on causal insights from semi‐structured interviews conducted with experts in intergenerational practice. The purpose of the interviews was to understand, from their personal and professional experiences in the field, how intergenerational programmes ‘work’. This was done by addressing questions such as ‘Why do you think some intergenerational programmes work and not others?’ and ‘What key components are required to develop a successful intergenerational programme?’ These questions were designed to help identify context, mechanisms, outcomes and their relationships, to inform our initial hypotheses and theory development. Ten experts were involved, including intergenerational practitioners, academics and older adult participants. Deakin University Research Ethics Committee provided ethical approval to conduct the interviews (project ID: 2023‐005).

#### 5.1.2 Stage 2: Search Strategy Design

The next step, from which point this protocol provides a prospective description of the realist review plan, is to seek empirical evidence to populate the theoretical framework, to either support, contradict or modify the programme theories (Pawson et al. 2005). A health sciences librarian from Deakin University (author RN) was involved in developing the search strategy for this review. In this stage, the following search terms and variations thereof will be used: ‘intergenerational programs’, ‘older adults’ and ‘preschool children’. The following seven electronic databases will be searched for peer‐reviewed literature: PsycINFO, SocINDEX, CINAHL, MEDLINE, ERIC, Academic Search Complete and Web of Science. Google Scholar will be searched using the same search terms to supplement the results. Google will be used to search for unpublished grey literature limited to organisational (.org) and governmental (.gov) websites. Forward citation searching will be performed on all relevant articles (which meet the inclusion criteria). Sources published in the English language from the year 2000 will be included. The justification for this scope is twofold: first, most of the relevant literature reporting empirical studies with outcomes were published after 2000, compared to more descriptive publications earlier (determined from literature review), and second, as a practical way to narrow the scope of this rapid review. A sample search strategy can be found in Supporting Information S2.

In line with the realist approach, additional searches may be undertaken at later stages, if the main search does not generate sufficient data to test programme theories or in response to potential programme theory refinements (Booth et al. 2019; Wong et al. 2013).

##### 5.1.2.1 Inclusion Criteria

No limits will be applied to the study designs of included research articles or any other document type that informs the review, including non‐empirical study sources or grey literature. This is because, for realist reviews, the value of sources does not lie in their design or methodology but in the extent to which their content and findings contribute to the testing of IPTs (Wong et al. 2013). The PICO (Population, Intervention, Comparator, Outcome) framework is adopted to describe the review inclusion criteria (Table 2). The Population of interest is older adults aged 65 years and over, or 50 and over for Aboriginal and Torres Strait Islander peoples, who reside in aged care settings or the community. Residential aged care includes settings that provide 24‐h supervision and/or care assistance for older people aged 65 years and over (or 50 years and over for Aboriginal and Torres Strait Islander peoples). Whilst this review is focused on IGPs in residential aged care, only a few articles on IGPs involving preschool children in institutional aged care settings were identified in the authors' scoping and test searches. Thus, articles on IGPs in community settings and involving older adults who do not live in residential aged care (e.g., community and volunteer groups, service providers, councils, etc.) will also be included in the screening process to identify any relevant information that may contribute to the programme theories. The Intervention is non‐familial intergenerational programmes; programmes where the intergenerational aspect is incidental and not planned or purposeful will not be included. There is no comparator for this review. The primary outcomes of interest are measures of psychosocial well‐being in older adult participants. This includes three categories of outcomes: psychological, emotional and social (Table 3).

**Table 2:** Inclusion and exclusion criteria (PICO framework).

Inclusion criteria	
Population	Older adults (65+ years); AND Preschool children (i.e., early childhood learning centre, childcare, playgroups)
Intervention	Non‐familial intergenerational programme
Comparator	None
Outcomes	Any measure of psychosocial health and/or well‐being
Study type	Quantitative, qualitative and mixed methods. All published study types reporting empirical research. Non‐empirical study sources (i.e., grey literature) will also be included, such as opinion papers, guidelines and dissertations.

Exclusion criteria	
• Familial intergenerational programmes.	
• Programmes where the intergenerational aspect is incidental and not planned nor purposeful.	
• Programmes with samples of adults < 65 years AND/OR school‐aged children.	
• Programmes focused on older adults with a diagnosis of dementia.	
• Non‐English sources.	
• Sources published before 2000.	

**Table 3:** Review outcome (psychosocial well‐being of older adults) including specific definitions and examples of outcome measures.

	Component/aspect	Definition	Examples of validated outcome measures
**Outcome: Psychosocial Well‐being of older adults**	**Psychological** (Ryff's Model of Psychological Well‐being 1989; Erikson's stages of psychosocial development, 1950)		
	Autonomy	Extent of self‐determination and independence; level of resistance to social pressures; internal locus of control (regulation of behaviour from within)	Ryff's Scales of Psychological Well‐being (PWBC; Ryff 1989)
	Environmental mastery	Sense of competence in managing the environment and everyday affairs; capacity to control array of external activities; sense of control over the external world to suit personal needs and values.	PWBC (Ryff 1989)
	Personal growth	Feelings of continued development versus stagnation; interest in life; openness to new experiences; and sense of improvement and realising of one's potential.	PWBC (Ryff 1989) Personal Growth Initiative Scale (PGIS; Robistschek 1998)
	Purpose in life	Directedness and meaning in life; outlook; beliefs that give life purpose; and aims and objectives for living.	PWBC (Ryff 1989) Life Engagement Test (Scheier et al. 2006)
	Self‐acceptance	Attitudes towards oneself and one's past life; acknowledgement and acceptance of multiple aspects of self, including good and bad qualities.	PWBC (Ryff 1989) Rosenberg Self‐Esteem Scale (RSES; 1965)
	Positive relations with others	Presence of warm, satisfying, trusting relationships with others; capability for strong empathy, affection, intimacy and compromise in relationships.	PWBC (Ryff 1989)
	Self‐confidence	Belief in oneself of being capable of successfully meeting demands of a task (broadly, life's challenges).	Trait Robustness of Self‐Confidence (TROSCI; Beattie et al. 2011) Personal Evaluation Inventory (PEI; Shrauger and Schohn 1995)
	Generativity	Interest in and dedication to benefitting future generations (Erikson 1963); inner desire and concern for the next generation.	Loyola Generativity Scale (LGS; McAdams and de St Aubin 1993) Generativity Scale (Ryff and Heincke 1983)
	**Social** (Keyes' Model of Social Well‐being 1998)		
	Social integration	Evaluation of the quality of one's relationship with the society and community.	Social Well‐Being Scale (Keyes 1998)
	Social acceptance	Trusting others, holding positive opinions about others.	Social Well‐Being Scale (Keyes 1998)
	Social contribution	Evaluation of one's social value, including the belief that one is a vital member of society with something of value to give to the world.	Social Well‐Being Scale (Keyes 1998)
	Social adjustment	Combination of satisfaction with relationships, performance in social roles and adjustment to one's environment.	Social Adjustment Scale Self‐Report (SAS‐SR; Weissman 1999) Social Functioning Questionnaire (SFQ; Tyrer et al. 2005)
	Social support	Number of contacts in one's social network, and satisfaction with those contacts; availability of people one trusts, can rely on and feel cared for and valued as a person.	Social Support Questionnaire (SSQ; Sarason et al. 1983)
	**Emotional**		
	Mood		Geriatric Depression Scale (GDS; Yesavage and Sheikh 1986) Profile of Mood States (POMS; Gibson 1997)

#### 5.1.3 Stage 3: Document Selection and Appraisal

Retrieved articles will be imported into Endnote, where duplicates will be removed. Using the Covidence online tool, the selection process will begin with the title and abstract screening. A title and abstract form will be pre‐tested by two reviewers for at least 30 abstracts (Garritty et al. 2021). Once agreement is reached, two reviewers will independently screen the remaining abstracts. A third reviewer will resolve any disagreements or conflicts.

The final article selection will rely on a comprehensive reading of the documents. A full‐text screening form (see Supporting Information S4) including details such as article number, reference, source database, inclusion or exclusion reason(s) and quality assessment will be developed and tested by two reviewers using 5 to 10 of the same articles (Rapin et al. 2022). Once piloted and calibrated, author 1 will screen all remaining full‐text articles. Two reviewers will independently review as a second reviewer at this stage. Any disagreements will be discussed with a third reviewer to reach a consensus. Reasons for inclusion or exclusion will be recorded in Covidence, where reviewers can work concurrently but independently.

##### 5.1.3.1 Quality Assessment

Quality assessment of selected studies will be guided by the Realist and Meta‐narrative Evidence Syntheses: Evolving Standards II (RAMESES II) criteria and publication standards (Wong et al. 2013), as well as the work by Dada and colleagues (Dada et al. 2023). The RAMESES standards (Wong et al. 2013) recommend that realist reviews include sources based on: (1) Relevance to theory and (2a) Rigour in terms of credibility and trustworthiness to determine fit for purpose. This will be assessed using the Mixed Methods Appraisal Tool (MMAT; Hong et al. 2018) for primary sources and the Critical Appraisal Skills Programme (CASP) Systematic Reviews Checklist (2018) for secondary sources. Dada et al. (2023) further propose that (2b) the rigour of resources be assessed in terms of coherence of theory, that is, is the theory consilient, simple and analogous to substantive theory? An additional criterion, (3) Richness, will also be appraised, assessing how meaningfully the resource can contribute to theory development or testing *(*Dada et al. 2023). Richness addresses whether the data source can meaningfully contribute to theory development or testing. It will be assessed in two ways. First, in relation to its conceptual richness, as rich, thick, or thin. Second, richness will also be assessed in relation to the review's research question, from a scale of ‘0’ (nothing of interest to the research question; not focused on design, implementation or use) to ‘4’ (much valuable data). A table outlining the above‐described quality appraisal criteria is provided in Supporting Information S5.

#### 5.1.4 Stage 4: Data Extraction

Data extraction will occur in a twofold process. First, a purpose‐built data extraction form (see Supporting Information S6) will be tested by two reviewers on a 10% selection of the included articles and modified as necessary. Author 1 will then extract data from the remaining articles, and other team members will check the results. This form will include the following categories: bibliographic details, aims and methods, participants, intervention details (intergenerational programme design, delivery, setting or context), findings and specific contributions to IPTs (Simionato et al. 2023).

The second stage of data extraction involves uploading the sources to Lumivero NVivo 15 software for coding. Data will be coded (a) deductively using the IPTs generated; (b) inductively using the data that emerge from the selected sources; and (c) retroductively, where potential causal processes or context‐sensitive mechanisms in relation to the desired outcomes are identified. Consistency checks on 20% of the coded documents will be performed between authors 1 and 2, with any disagreements or conflicts resolved through discussions with a third reviewer. If disagreement is high (i.e., 20% and above), an incremental 10% of sources will be checked until high agreement (i.e., 80% and above) is achieved.

#### 5.1.5 Stage 5: Analysis and Synthesis

Data analyses will centre on examining the causal connections between context and mechanisms that offer compelling insights into the specified outcome (i.e., impacts on the psychosocial well‐being of older adults). To test explanatory leads, Wong (2018) suggests two criteria rooted in abductive reasoning within the context of realist epistemology: plausibility and consistency. Plausibility is characterised as the most plausible explanatory theory based on current knowledge. The following criteria will be utilised to evaluate the consistency of potential programme theories: (i) consilience, which assesses the theory's ability to explain the most data; (ii) simplicity, indicating that the theory is straightforward and does not require other ‘ad hoc’ assumptions to account for the data; and (iii) analogy, ensuring that the theory aligns with existing knowledge (Wong 2018).

Explanatory accounts will be constructed from the gathered data and the impact of those factors on reported mechanisms and programme outcomes. Subsequently, this collection of explanatory narratives will be subjected to iterative analysis to identify patterns of occurrence or demi‐regularities. Author 1 will independently code the data using NVivo software. Emerging insights will be shared and discussed with the review team. The identified patterns of occurrence will then be amalgamated into thematic clusters, forming a context–mechanism–outcome configuration for each thematic cluster.

#### 5.1.6 Stage 6: Presentation of Revised Theory

The refined and revised programme theory will be presented to IGP experts interviewed in Stage 1 for consultation, inviting their input on the content and presentation of theory. Findings will be shared with stakeholders from an aged care organisation and early childhood learning centre who have committed to involvement in a subsequent study of an IGP. The results will also be published in a peer‐reviewed journal and presented at relevant conferences. Finally, this groundwork will inform the realist evaluation of one of Australia's first intergenerational shared‐roof facilities.

## Author Contributions

- **Content:** Lysha Z. Y. Lee is ideally suited for the role of content expert (intergenerational programmes). She has a background in psychology and has been involved in the design, implementation and evaluation of intergenerational programmes in various settings since 2021. Alison M. Hutchinson and Katrin Gerber also have backgrounds and content expertise in older adult and aged care research. Katrin Gerber's research also specialises in the fields of ageing and mental health.
- **Review methods:** Professor Alison M. Hutchinson brings expertise in realist synthesis. She co‐authored two highly cited peer‐reviewed publications in the field (McCormack et al. 2013; Rycroft‐Malone et al. 2012) as well as a book chapter (Rycroft‐Malone et al. 2015). In addition, Professor Alison M. Hutchinson has authored two other realist reviews and multiple systematic reviews. Professor Alison M. Hutchinson also brings extensive experience in undertaking research in the residential aged care setting.
  Associate Professor Patricia Nicholson has extensive experience and expertise in conducting systematic reviews, with 5 published systematic reviews. She is a Senior Fellow of the Higher Education Academy, a nationally respected clinician, academic and researcher and is recognised as an expert in higher education.
  Dr. Katrin Gerber has conducted systematic reviews in the fields of ageing and mental health, including a supervised Master's thesis involving a systematic review on grief in older adults and health service use.
- **Statistical analysis:** Professor Alison M. Hutchinson is experienced in realist review analysis methods. Dr. Katrin Gerber has expertise in mixed methods, including qualitative, quantitative and arts‐based methods as well as narrative synthesis. Lysha has received training in realist analysis and coding methods.
- **Information retrieval:** Ramona Naicker, a STEMM Scholarly Services Librarian at Deakin University, brings extensive experience in systematic reviews and evidence‐based practice. Previously, she led the evidence service at a UK NHS library, where she supported systematic review searches and developed search strategies for organisations such as Public Health England and Age UK. With her expertise in information retrieval and experience in supporting complex reviews, Ramona has played a key role in developing the search strategy for this review.

## Supporting information

Supporting information 1: PRISMA‐P checklist.

Supporting information 2: Sample search strategy.

Supporting information 3: Sequence of stages of the rapid realist review.

Supporting information 4: Full text screening form.

Supporting information 5: Quality appraisal criteria.

Supporting information 6: Data extraction form.
