# Supplementary material for: PROTOCOL: Understanding Intergenerational Programmes to Improve the Psychosocial Health and Well‐Being of Older Adults in Residential Aged Care: A Rapid Realist Review Protocol
Source: Campbell Syst Rev. 2025 Apr 8;21(2):e70023. doi: 10.1002/cl2.70023 (PMC11976665; doi:10.1002/cl2.70023)
Supplement: Supplementary file 2 — Supporting information 2: Sample search strategy. [file CL2-21-e70023-s005.docx]

## **Supporting Information 2**

## **Sample search strategy:** Medline Complete (EBSCOhost)

| **#** | **Searches** |
| --- | --- |
| S1 | TI Intergeneration* N3 program* OR AB Intergeneration* N3 program* |
| S2 | TI Intergeneration* N3 intervention* OR AB intervention * N3 program* |
| S3 | TI Intergeneration* N3 activit* OR AB intervention * N3 activit* |
| S4 | TI Intergeneration* N3 practice* OR AB intervention * N3 practice* |
| S5 | TI Intergeneration* N3 project* OR AB intervention * N3 project* |
| S6 | TI Intergeneration* N3 relation* OR AB Intergeneration* N3 relation* |
| S7 | (MH “Intergenerational Relations”) |
| **S8** | **S1 OR S2 OR S3 OR S4 OR S5 OR S6 OR S7** |
| S9 | TI elder* OR AB elder* |
| S10 | TI senior* OR AB senior* |
| S11 | TI aged* OR AB aged* |
| S12 | TI old* N3 adult* OR AB old* N3 adult* |
| S13 | TI old* N3 person* OR AB old* N3 person* |
| S14 | TI old* N3 people* OR AB old* N3 people* |
| S15 | (MH “Aged+”) |
| S16 | (MH “Aged, 80 and over+” |
| **S17** | **S9 OR S10 OR S11 OR S12 OR S13 OR S14 OR S15 OR S16 OR S17** |
| S18 | TI Preschool* OR AB Preschool* |
| S19 | TI “Pre-school*” OR AB “Pre-school*” |
| S20 | TI Kindergarten* OR AB Kindergarten* |
| S21 | TI "young* child*" OR AB "young* child*" |
| S22 | TI nursery OR AB nursery |
| S23 | TI nurseries OR AB nurseries |
| S23 | (MH "Child, Preschool") |
| **S24** | **S18 OR S19 OR S20 OR S21 OR S22 or S23** |
| S25 | S8 AND S17 AND S24 |
| S26 | S8 AND S17 AND S24 (Date: 20000101-20241231; English Language) |
