# Supplementary material for: PROTOCOL: Understanding Intergenerational Programmes to Improve the Psychosocial Health and Well‐Being of Older Adults in Residential Aged Care: A Rapid Realist Review Protocol
Source: Campbell Syst Rev. 2025 Apr 8;21(2):e70023. doi: 10.1002/cl2.70023 (PMC11976665; doi:10.1002/cl2.70023)
Supplement: Supplementary file 3 — Supporting information 3: Sequence of stages of the rapid realist review. [file CL2-21-e70023-s004.docx]

## **Supporting Information 3**

Review outcome (psychosocial well-being of older adults), including specific definitions and examples of outcome measures

| **Outcome: Psychosocial Well-being (older adults)** | **Component/ Aspect** | **Definition** | **Example of validated outcome measures** |
| --- | --- | --- | --- |
|  | **Psychological** (Ryff’s Model of Psychological Well-being, 1989; Erikson’s stages of psychosocial development, 1950) | | |
|  | Autonomy | Extent of self-determination and independence; level of resistance to social pressures; internal local of evaluation (regulation of behaviour from within) | Ryff’s Scales of Psychological Well-being (PWBC; Ryff, 1989) |
|  | Environmental mastery | Sense of competence in managing the environment and everyday affairs; capacity to control an array of external activities; a sense of control over the external world to suit personal needs and values | PWBC (Ryff, 1989) |
|  | Personal growth | Feelings of continued development vs stagnation; interest in life; openness to new experiences; a sense of improvement and realising one’s potential | PWBC (Ryff, 1989)  Personal Growth Initiative Scale (PGIS; Robistschek, 1998) |
|  | Purpose in life | Directedness and meaning in life; outlook; beliefs that give life purpose; aims and objectives for living | PWBC (Ryff, 1989)  Life Engagement Test (Scheier et al., 2006) |
|  | Self-acceptance | Attitudes towards oneself and one’s past life; acknowledgement and acceptance of multiple aspects of self, including good and bad qualities | PWBC (Ryff, 1989)  Rosenberg Self-Esteem Scale (RSES; 1965) |
|  | Positive relations with others | Presence of warm, satisfying, trusting relationships with others; capability for strong empathy, affection, intimacy, and compromise in relationships | PWBC (Ryff, 1989) |
|  | Self-confidence | Belief in oneself of being capable of successfully meeting the demands of a task (broadly, life’s challenges) | Trait Robustness of Self-Confidence (TROSCI; Beattie et al., 2010)  Personal Evaluation Inventory (PEI; Shrauger & Schohn, 1995) |
|  | Generativity | Interest in and dedication to benefitting future generations (Erikson, 1963; McAdams, 2006); inner desire and concern for the next generation | Loyola Generativity Scale (LGS; McAdams & de St Aubin, 1992)  Generativity Scale (Ryff & Heincke, 1983) |
|  | **Social** (Keyes’ Model of Social Well-being, 1998; Larson’s Model of Social Well-being, 1992) | | |
|  | Social integration | Evaluation of the quality of one’s relationship to society and community | Social Well-Being Scale (Keyes, 1998) |
|  | Social acceptance | Trusting others, holding positive opinions about others | Social Well-Being Scale (Keyes, 1998) |
|  | Social contribution | Evaluation of one’s social value, including the belief that one is a vital member of society with something of value to give to the world | Social Well-Being Scale (Keyes, 1998) |
|  | Social adjustment | Combination of satisfaction with relationships, performance in social roles, and adjustment to one’s environment | Social Adjustment Scale Self-Report (SAS-SR; Weissman, 1999)  Social Functioning Questionnaire (SFQ; Tyer et al., 2005) |
|  | Social support | Number of contacts in one’s social network, and satisfaction with those contacts; availability of people one trusts, can rely on, and feel cared for and valued as a person | Social Support Questionnaire (SSQ; Sarason et al., 1983) |
|  | **Emotional** | | |
|  | Mood |  | Geriatric Depression Scale (GDS; Yesavage & Sheikh, 1986)  Profile of Mood States (POMS; Gibson, 1997) |
