# Supplementary material for: PROTOCOL: Understanding Intergenerational Programmes to Improve the Psychosocial Health and Well‐Being of Older Adults in Residential Aged Care: A Rapid Realist Review Protocol
Source: Campbell Syst Rev. 2025 Apr 8;21(2):e70023. doi: 10.1002/cl2.70023 (PMC11976665; doi:10.1002/cl2.70023)
Supplement: Supplementary file 4 — Supporting information 4: Full text screening form. [file CL2-21-e70023-s003.docx]

## **Supporting Information 4**

Full text screening form

| Full Text Screening Form | | |  |
| --- | --- | --- | --- |
| The aim of this realist review is to develop evidence-informed and operational theory or theories of what works, for whom, under what circumstance(s) and how intergenerational programs improve the psychosocial health and wellbeing of older adults in residential aged care. Specifically, the review seeks to develop and identify the following:   - Contexts and mechanisms that may facilitate or hinder the effectiveness of intergenerational programs (on psychosocial health and wellbeing of older adult participants); - The relationship between the above contexts and mechanisms, and how they lead to specific outcomes; - Refined theory or theories of how intergenerational programs work to improve psychosocial health and wellbeing of residents in residential aged care settings. | | |  |
| **Population** | **Include** | **Exclude** |  |
|  | - Older adults aged 65 years and over, OR 50 and over for Aboriginal and Torres Strait Islander peoples - Preschool aged children, including those attending childcare or early learning centres (or equivalent), playgroups, etc | - Adults younger than 65 years, or younger than 50 years for Aboriginal and Torres Strait Islander people - Focused only on adults with dementia - Young people school-aged and older (e.g., students, teenagers) |  |
| **Intervention** | **Include**   - Articles that describe the implementation and/or evaluation of non-familial intergenerational program(s) and/or practice, where younger and older participants are not related | **Exclude**   - Articles that describe familial intergenerational program(s) and/or practice - Articles that describe program(s) with unintentional intergenerational aspects (i.e., not planned or purposeful) - Articles that briefly describe intergenerationality as a concept without further elaboration |  |
| **Outcome measures** | **Include**   - Articles for which impacts to the psychosocial health and/or well-being of older adult participants is an aim and/or outcome*   Also if:   - Includes relevant theory - Includes discussion with explanatory value to RQ/ program theory | **Exclude**   - Articles for which impacts to older adult participants is not an aim and/or outcome - No relevant theory, outcomes, nor discussion with explanatory value |  |
| **Language** | **Include**   - Articles published in the English language | **Exclude**   - Articles published in a language other than English |  |
| **Publication date** | **Include**   - Articles published in and after 2000 | **Exclude**   - Articles published before 2000 |  |
