# Supplementary material for: PROTOCOL: Understanding Intergenerational Programmes to Improve the Psychosocial Health and Well‐Being of Older Adults in Residential Aged Care: A Rapid Realist Review Protocol
Source: Campbell Syst Rev. 2025 Apr 8;21(2):e70023. doi: 10.1002/cl2.70023 (PMC11976665; doi:10.1002/cl2.70023)
Supplement: Supplementary file 5 — Supporting information 5: Quality appraisal criteria. [file CL2-21-e70023-s002.docx]

## **Supporting Information 5**

Quality appraisal criteria

| **Quality Appraisal** | |
| --- | --- |
| **Relevance**  *Does the resource contain data relevant to the topic area/ program theories?* | High relevance   - Draws upon a theoretical explanation for the effectiveness of IGPs in the literature review. - Relates to older adults who have participated in IGP(s) involving preschool children, and includes detailed demographic characteristics of participants. - Includes an in-depth description of the IGP (e.g., activities, facilitators, duration, frequency) and its implementation process. - Describes the context and setting the IGP is implemented in, including motivations behind implementation. - Reports the outcomes of the IGP(s), including detailed analysis and changes to various measures of psychosocial health and wellbeing in older adult participants. - Includes in-depth explanation for reported outcomes, including various levels of explanation (e.g., psychological, social, biological) and drawing upon existing literature/ theory. |
|  | Moderate relevance   - Relates to older adults who have participated in IGP(s) involving preschool children and includes basic description of participants’ demographic characteristics (e.g., age). - Includes a description of the IGP (e.g., activities, facilitators, duration, frequency). - Describes the context and setting the IGP is implemented in. - Reports the outcomes of the IGP(s), highlighting at least one measure of psychosocial health or wellbeing in older adult participants. - Includes explanation for and discussion of reported outcomes, briefly drawing upon existing literature/ theory. |
|  | Low relevance   - Relates to older adults who have participated in IGP(s) involving preschool children. - Includes a brief description of the IGP, including context and setting the IGP is implemented in. - Briefly reports the outcomes of the IGP, but not as a focus of the article. - Briefly discusses reported outcomes but does not draw upon existing literature/ theory. |
|  | No relevance – does not meet any of the above criteria. |
| **Richness**  *Can the resource meaningfully contribute to theory development or testing?* | Concepts [1]   - Conceptually rich: articles with well-grounded and clearly described theories and concepts. - Conceptually thick: articles with a rich description of a progran is provided, but without explicit reference to the theory underpinning it. - Conceptually thin: studies with weak program descriptions where discerning theory would be problematic |
|  | In relation to RQs [2]  0 = nothing of interest, not focused on design, implementation or use  1 = limited data of interest, likely to appear in other articles  2 = limited data of interest, but quick to extract it and could add weight to finding  3 = some good quality data  4 = much valuable data |
| **Rigour** – trustworthiness  *Is the source credible? Are the methods used appropriate and trustworthy?* | Mixed Methods Appraisal Tool (MMAT) [3] |
| **Rigour** – coherence of theory  *Is the theory consilient?*  *Is the theory simple?*  *Is the theory analogous to substantive theory?* | Consilient?  A consilient theory explains a wide range of data and observations; that the theory can account for and integrate diverse pieces of evidence, providing a comprehensive explanation for the phenomenon under study. A consilient theory would effectively explain various Context-Mechanism-Outcome configurations (CMOCs) identified during the review process.   - Yes - No |
|  | Simple?  A simple theory makes few assumptions. Simplicity refers to the theory's ability to explain complex interventions or phenomena without relying on numerous or convoluted explanations. A simple theory is more accessible and easier to apply across different contexts.   - Yes - No |
|  | Analogous to substantive theory?  This attribute means that the developed theory aligns with existing credible theories in the field. It should be compatible with and build upon established knowledge, rather than contradicting well-supported theoretical frameworks.   - Yes - No |
| [1] Calderon-Larrañaga S, Milner Y, Clinch M, Greenhalgh T, Finer S. Tensions and opportunities in social prescribing. Devel-oping a framework to facilitate its implementation and evaluation in primary care: a realist review. BJGP Open. 2021;5(3).  [2] Waldron C, Cahill J, Cromie S, et al. Personal electronic Records of Medications (PERMs) for medication reconciliation at care transitions: a rapid realist review. BMC Med Inform Decis Mak. 2021;21(1):307.  [3] Hong QN, Pluye P, Fàbregues S, Bartlett G, Boardman F, Cargo M, Dagenais P, Gagnon M-P, Griffiths F, Nicolau B, O’Cathain A, Rousseau M-C, Vedel I. Mixed Methods Appraisal Tool (MMAT), version 2018. Registration of Copyright (#1148552), Canadian Intellectual Property Office, Industry Canada | |

Adapted from Dada, S., Dalkin, S., Gilmore, B., Hunter, R., & Mukumbang, F. C. (2023). Applying and reporting relevance, richness and rigour in realist evidence appraisals: Advancing key concepts in realist reviews. Research synthesis methods, 14(3), 504-514.
