# Supplementary material for: PROTOCOL: Understanding Intergenerational Programmes to Improve the Psychosocial Health and Well‐Being of Older Adults in Residential Aged Care: A Rapid Realist Review Protocol
Source: Campbell Syst Rev. 2025 Apr 8;21(2):e70023. doi: 10.1002/cl2.70023 (PMC11976665; doi:10.1002/cl2.70023)
Supplement: Supplementary file 6 — Supporting information 6: Data extraction form. [file CL2-21-e70023-s001.docx]

## **Supporting Information 5**

Data extraction tool

| **Bibliographic details** | |
| --- | --- |
| Article number/ ID: |  |
| Article reference: |  |
| Geographical details of the study |  |
| Study funding source(s) |  |
| **Aims & Methods** | |
| What is the study type? |  |
| Methods used? |  |
| Aims and objectives clearly stated? |  |
| Is the research question clearly stated? |  |
| How was the data analysed? |  |
| Does the intervention use a particular theory to inform its design |  |
| **Participants** | |
| What is the sample size? |  |
| What is the sample characteristics? |  |
| Age range of older adults |  |
| Age range of children |  |
| How were participants recruited? |  |
| What were the inclusion and exclusion criteria? |  |
| **Intervention details** | |
| What was the intervention? |  |
| Were the aims/ objectives of the program reported? |  |
| How was the intervention delivered? |  |
| Who delivered the intervention? |  |
| In what setting was the intervention delivered? (Why was this context chosen?) |  |
| How long and how frequent was the intervention |  |
| Was the intervention designed with the participants (one or both age groups)? |  |
| **Findings/ Results** | |
| What data was collected? |  |
| What were the reported experiences of participants? |  |
| What were the reported experiences of others (eg facilitators)? |  |
| Did the intervention focus on/ impact on the psychosocial well-being of older adults? |  |
| What worked? *i.e. facilitators, if reported (e.g., facilitators, activities, structure, etc)* |  |
| What didn’t work? *i.e. challenges and barriers, if reported* |  |
| Are the findings interpreted within the contexts of other studies and theory? |  |

Theory

| **IPT 1: A safe space and time to develop meaningful connections and trust** | | | |
| --- | --- | --- | --- |
| CONTEXT | MECHANISM-RESOURCE | MECHANISM-RESPONSE | OUTCOMES |
| Older adults without ‘organic’ exposure to and thus do not typically get to meet and interact with preschool children in their lives (familial or otherwise). | Opportunity to meet and share experiences with preschool children in a safe space through shared activities, and over time (not once off). | Develop shared interests; Develop meaningful connections and relationships | Increase companionship; Positive and meaningful social connections; Feel seen and heard by children |
|  |  |  |  |
|  | | | |
| 1b (rival theory) |  |  |  |
|  |  | Fail to bond and develop positive relationship with preschool children, e.g., if unable to communicate effectively or meet one another’s needs. | Negative mood (eg frustration, depression) from persistent negative experiences; negative attitude towards intergenerational activities/ relationships. |
|  | | | |

| **IPT 2: Achieving generativity through developmentally congruent social roles** | | | |
| --- | --- | --- | --- |
| CONTEXT | MECHANISM-RESOURCE | MECHANISM-RESPONSE | OUTCOMES |
| Loss of contributory roles and becoming a passive recipient of help/ care (being in assisted living facilities; health decline). | Opportunity to step into an active role where they can contribute/ share/ impart knowledge and/or skills to the younger generation, and an acknowledgement of this by others . | Feelings of fulfilment; Feel valued through role fulfilled; Sense of dignity; Challenge self-directed ageism | Achieving generativity; Increased purpose in life congruent to their stage of life; Increased confidence and self-esteem |
| Not reported on. |  |  |  |
| 2b (rival theory) |  |  |  |
|  |  | Reminder of abilities/ roles lost, especially if compared with more ‘able’ participants. | Feelings of despair/disappointment and reduced confidence and self-esteem. |
|  |  |  |  |

| **IPT 3: Empowerment and mastery through lifelong learning** | | | |
| --- | --- | --- | --- |
| CONTEXT | MECHANISM-RESOURCE | MECHANISM-RESPONSE | OUTCOMES |
| Loss of contributory roles and becoming a passive recipient of help/ care (being in assisted living facilities; health decline). | A loss of (previous) roles and opportunities for achievement, alongside ageing and moving to a residential aged care facility. | Opportunity to (re)learn skills and knowledge through activities appropriate to their capability levels (cognitive, physical, etc.) and interests, with preschool children; Provision of assistance/ guidance/ ‘scaffolding’, when needed and requested | Motivation of self-improvement;  Sense of mastery and accomplishment; Development of mutual respect;  Challenge self-directed ageism |
|  |  |  |  |
| 3b (rival theory) |  |  |  |
|  |  | Overwhelm experienced from learning expectations; Reminder of abilities/ roles lost (especially if activities are not appropriate) | Disappointment and reduced confidence and self-esteem. |
|  |  |  |  |

| **IPT 4: Autonomy, agency and collaboration** | | | |
| --- | --- | --- | --- |
| CONTEXT | MECHANISM-RESOURCE | MECHANISM-RESPONSE | OUTCOMES |
| ‘Typical’ loss of (previous) roles; Many activities in RAC settings are pre-determined and do not involve residents in decision-making about the activities (?) | Opportunity to collaboratively co-design a program, allowing residents to incorporate personal interests and needs; Flexibility for different levels of participation | Sense of agency & autonomy; Vested interest and engagement; Sense of belonging; Program acceptability from residents' POV | Autonomous, meaningful participation & engagement; Reduced program attrition; Increased purpose |
|  |  |  |  |
| 4b (rival theory) |  |  |  |
|  |  | Friction from failure to coordinate collaboration efforts and compromise | Incohesive program aims and design, leading to poor engagement and program attrition |
|  |  |  |  |

| **IPT 5: Feeling more ‘at home’ through improved relationships with aged care staff** | | | |
| --- | --- | --- | --- |
| CONTEXT | MECHANISM-RESOURCE | MECHANISM-RESPONSE | OUTCOMES |
| Care home staff are people that aged care residents likely interact with the most, in their 'home' – the nature of their relationships naturally affects their daily experiences. Typically, the dynamic is limited to one of ‘carer’ and ‘recipient’ (resident). | Opportunity and time for aged care staff to get to know residents on a deeper level, learning about their lives and who they are as individuals (who they were before moving into the RACF), to see beyond their care needs (what staff are predominantly focused on). | Aged care staff and care providers may develop more positive perceptions of residents, seeing them more holistically as an individual (versus someone with the care needs they provide). | More positive experience of living in aged care home, supported by deeper and more meaningful relationships (with aged care staff). |
|  |  |  |  |
| 5b (rival theory) |  |  |  |
|  |  |  |  |

| **IPT 6: Environment as a third teacher** | | | |
| --- | --- | --- | --- |
| CONTEXT | MECHANISM-RESOURCE | MECHANISM-RESPONSE | OUTCOMES |
| Participants' varied associations with the residential aged care facility, including specific spaces used for IG activities. | A space, or elements in the space, designed in a way that encourages/ guides/ directs intergenerational encounters (e.g., placement of furniture, or having to share materials). | Participants are 'guided' and ‘encouraged’ by the environment to interact and engage with one another; Increased intergenerational interactions | Development of intergenerational connections and relationships |
|  |  |  |  |
| 6b (rival theory) |  |  |  |
|  |  |  |  |

| **IPT 7: Skilled facilitators as a conduit** | | | |
| --- | --- | --- | --- |
| CONTEXT | MECHANISM-RESOURCE | MECHANISM-RESPONSE | OUTCOMES |
| Not all older adults will know 'how' to engage with preschool children (and vice versa);  Need specific skills to facilitate intergenerational programs | Provision of skilled facilitators (staff/ volunteers) who support the interactions between the younger and older participants (i.e., striking a balance between facilitating and stepping back to allow the participants to lead and for organic interaction to occur) = source of scaffolding and guidance. | Risks are managed, with safeguards put in place; Education for participants (role expectations, rules of engagement, social norms); Participant anxiety reduced; Spontaneous and organic connections and interactions can occur. | Safe and supported place where intergenerational relationships can be fostered, and risks/ negative outcomes can be mitigated. |
|  |  |  |  |
| 7b (rival theory) |  |  |  |
|  |  |  |  |

| **IPT 8: Organisational leadership** | | | |
| --- | --- | --- | --- |
| CONTEXT | MECHANISM-RESOURCE | MECHANISM-RESPONSE | OUTCOMES |
| Organisations involved (childcare, aged care) typically operate in silo, and there is not integration in terms of policies and operation. | Establishment of shared understanding and vision/purpose/goal(s) between organisations of how to help one another; Sharing of resources between organisations (to achieve said goals); Communication pathway/ platform | Allocated resources to execute IGP, and meet all other conditions as per previous CTs and to achieve program success (e.g. staff training, time, transportation, funding, etc); Buy-in, collaboration and support amongst staff | Successful execution of intergenerational program as designed, increasing likelihood of achieving goals; Resources and support to manage setbacks to keep program ‘on track’, ultimately to enhance well-being of residents (amongst other goals). |
|  | | | |
